# A log-likelihood-gain intensity target for crystallographic phasing that accounts for experimental error

**DOI:** 10.1107/S2059798315013236

**Published:** 2016-03-01

**Authors:** Randy J. Read, Airlie J. McCoy

**Affiliations:** a Department of Haematology, University of Cambridge, Wellcome Trust/MRC Building, Hills Road, Cambridge CB2 0XY, England

**Keywords:** intensity-measurement errors, likelihood

## Abstract

The crystallographic diffraction experiment measures Bragg intensities; crystallo­graphic electron-density maps and other crystallographic calculations in phasing require structure-factor amplitudes. If data were measured with no errors, the structure-factor amplitudes would be trivially proportional to the square roots of the intensities. When the experimental errors are large, and especially when random errors yield negative net intensities, the conversion of intensities and their error estimates into amplitudes and associated error estimates becomes nontrivial. Although this problem has been addressed intermittently in the history of crystallographic phasing, current approaches to accounting for experimental errors in macromolecular crystallography have numerous significant defects. These have been addressed with the formulation of LLGI, a log-likelihood-gain function in terms of the Bragg intensities and their associated experimental error estimates. LLGI has the correct asymptotic behaviour for data with large experimental error, appropriately downweighting these reflections without introducing bias. LLGI abrogates the need for the conversion of intensity data to amplitudes, which is usually performed with the French and Wilson method [French & Wilson (1978 ▸), *Acta Cryst.* A**35**, 517–525], wherever likelihood target functions are required. It has general applicability for a wide variety of algorithms in macromolecular crystallography, including scaling, characterizing anisotropy and translational noncrystallographic symmetry, detecting outliers, experimental phasing, molecular replacement and refinement. Because it is impossible to reliably recover the original intensity data from amplitudes, it is suggested that crystallographers should always deposit the intensity data in the Protein Data Bank.

## Abstract 2

A new Rice-function approximation for the effect of intensity-measurement errors improves the treatment of weak intensity data in calculating log-likelihood-gain scores in crystallographic applications including experimental phasing, molecular replacement and refinement.

## 1. Introduction   

For macromolecular crystallography, maximum-likelihood functions are required in order to account for the large model errors that are present during phasing. In this way, macromolecular crystallography differs from small-molecule crystallography, where the model errors are small and the most widely used and successful program for refinement, *SHELXL* (Sheldrick, 2015 ▸), uses a least-squares (intensity) target. Compared with the model errors, the relatively smaller data errors have not been the focus of the development of macromolecular likelihood functions, but recent advances have raised the importance of dealing properly with both large model and large data errors. Most prominently, it has been demonstrated that useful information can be extracted from very weak diffraction data (Ling *et al.*, 1998 ▸; Karplus & Diederichs, 2012 ▸). This has coincided with the uptake of photon-counting area detectors for macromolecular crystallography, on which data are frequently integrated beyond traditional resolution limits [for example, where the merged *I*/σ(*I*) > 2]. Lastly, structure solution is increasingly being attempted with pathologies such as twinning, high anisotropy and translational NCS (Read *et al.*, 2013 ▸). In the last two of these cases, weak data with high error cannot be excluded because they form an essential part of the analysis.

The sources of error in the measurement of intensities are reasonably well understood, and there are good arguments for assuming that these errors can be considered to be drawn from Gaussian probability distributions (even though the size of the errors can be hard to calibrate). Photon counting gives rise to Poisson distributions, which can be approximated reasonably well by Gaussian distributions, even for a few tens of counts. The estimation of peak intensities involves taking the difference between the counts in the peak area and the counts arising from background scattering, and the distribution of the difference between two random numbers drawn from Poisson distributions is approximated even better by a Gaussian. In addition, there are other sources of error, arising for instance from beam instability and uncertainties in detector calibration or the estimation of scale factors. As more sources of error accumulate, the central limit theorem tells us that the distribution of errors will tend more towards a Gaussian.

There are currently two conceptually disparate methods implemented for the incorporation of experimental errors into maximum-likelihood targets. The most widely used method is referred to here as ‘inflating the Rice variance’ (Green, 1979 ▸), while the other, far less frequently used method is the MLI target in *CNS* (Brünger *et al.*, 1998 ▸), originally called MLF2 (Pannu & Read, 1996 ▸). Both have strengths and serious deficiencies. In this work, we have aimed to remedy these deficiencies while preserving the strengths of both methods.

Leaving aside the effect of measurement error for the moment, current likelihood targets account for the model errors by considering that the phased differences between calculated and true structure factors arise from the sum of many small differences in the calculated and true contributions of the atoms making up the structure. By virtue of the central limit theorem, the relationship between the calculated and true phased structure factors can be approximated well with the complex normal distribution for acentric reflections or the real normal distribution for centric reflections. Since the phases are not measured in the diffraction experiment, likelihood targets require integrating over all possible phase choices for the acentric case, yielding the Rice function [equation 1*a* [Disp-formula fd1], derived first in the crystallographic context by Luzzati (1952 ▸), Sim (1959 ▸) and Srinivasan & Ramachandran (1965 ▸)] or summing over the two phase choices for the centric case (equation 1*b* [Disp-formula fd1]; Woolfson, 1956 ▸).
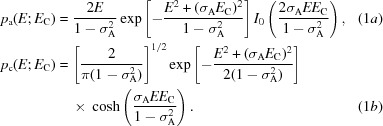



These equations are expressed in terms of normalized structure-factor amplitudes or *E* values for convenience in most of what follows. (The important terms used in the equations are summarized in Table 1 ▸.) σ_A_ is essentially the fraction of a calculated *E* value that is correlated with the true *E* value. Note that effects such as anisotropy or translational noncrystallographic symmetry can be accounted for in the computation of *E* values.

It is usual to express the likelihood in terms of a likelihood ratio, or a log-likelihood gain, which is the improvement (or otherwise) of the current model with respect to the null hypothesis (a random-atom or uninformative model). This is shown in (2), where the probability given an uninformative model, or no model, is the Wilson (1949 ▸) distribution, which can be obtained by setting σ_A_ in (1) to zero,
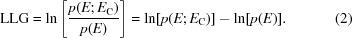



### 1.1. Inflating the Rice variance   

The ‘inflated-variance Rice’ method was originally introduced in the context of experimental phasing by single isomorphous replacement (Green, 1979 ▸), and it has subsequently been applied to both experimental phasing (de La Fortelle & Bricogne, 1997 ▸; McCoy *et al.*, 2004 ▸) and structure refinement (Murshudov *et al.*, 1997 ▸; Bricogne & Irwin, 1996 ▸). This approximation, given in (3*a*) for the acentric case and in (3*b*) for the centric case, is obtained from (1) by inflating the variance term (1 − σ_A_^2^) in the Rice functions.
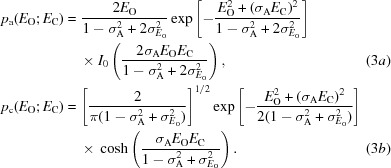



There are serious problems with the inflated-variance Rice-function approximation. Firstly, the derivation requires a chained series of approximations in which one statistical model is transformed into another. Inflating the Rice-function variance corresponds to a statistical model in which structure-factor errors are drawn (in the acentric case) from a complex Gaussian distribution. The resulting functional form is used to approximate the effect of Gaussian errors in the observed amplitudes. In turn, estimates of Gaussian errors in the observed amplitudes have to be obtained in some way from estimates of Gaussian errors in the observed intensities. While it is appropriate to assume that measurement errors for the intensities are drawn from a Gaussian distribution, the error distribution does not remain Gaussian after the transformation of intensities to amplitudes. Secondly, the inflated-variance Rice-function approximation requires a normalized amplitude *E*_O_ to be derived from the observed intensity, even when taking the difference between the peak measurement and the background may yield a negative net intensity. Thirdly, it requires a standard deviation for the normalized amplitude (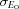) and a scale factor appropriate for the inflation of the variance (two in equation 3*a* [Disp-formula fd3] and one in equation 3*b* [Disp-formula fd3], as discussed below). Fourthly, the inflation of the variance breaks the normalization of the expected structure factors. Embedded in the attempts to solve these problems are a further series of complications.

When likelihood targets are formulated in terms of the structure-factor amplitudes rather than the intensities, the simplest approach for converting the intensities to amplitudes is to take the square root, after either discarding negative net intensities or setting them to zero, and to set the experimental amplitude errors using the first-order approximate formula 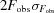 = 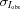. To avoid the asymptotic case as *F*_obs_ tends to zero, various improved functional forms have been proposed. Perhaps the most common is (4); this approximation is used in the program *ADDREF* (George Davenport & Syd Hall; http://www.iucr.org/__data/iucr/cif/software/xtal/xtal372htmlman/html/addref-desc.html), is implemented in the *cctbx* library (Grosse-Kunstleve *et al.*, 2002 ▸) and is equivalent to an option in the *CCP*4 program *TRUNCATE*.
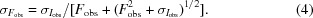
In a classic paper, French & Wilson (1978 ▸) introduced a Bayesian approach to the problem of structure-factor estimation from weak and even negative net intensity measurements, in which prior knowledge about the scattering power is combined with the experimental data to yield posterior distributions for the true amplitudes or intensities. From these posterior distributions, expected values and estimated standard deviations can be obtained for the true intensities or amplitudes. This approach is particularly valuable for one of its original purposes, *i.e.* to provide amplitudes that can be combined with phase information to compute electron-density maps and estimates of the experimental standard deviations for use in the least-squares refinement target functions that were available at the time.

The behaviour of the French and Wilson approach becomes problematic when the errors are large. As the intensity-measurement errors become larger, the posterior distributions come to be dominated by the prior Wilson distribution; in the limit of a measurement with no information content (infinite standard deviation for the intensity measurement), the posterior distribution is simply the Wilson distribution, which has a finite standard deviation. Thus, if the posterior distribution is interpreted as the result of an experimental measurement, an uninformative ‘measurement’ that should carry no weight in determining the details of the model ends up exercising significant influence on that model. Likelihood functions that account for experimental error should have the correct asymptotic behaviour as the integrated data fade to insignificance in the outer resolution shells. Ideally, including data with insignificant signal should just waste CPU time but should not affect the results.

In whatever way the scalar error in measuring an amplitude is derived from the intensity data, further errors are introduced in deriving the inflated-variance Rice distribution. For acentric reflections, the scalar error in measuring an amplitude is approximated as a complex error in the true structure factor, which is then used to increment the variance term in the Rice function (equation 1*a* [Disp-formula fd1]). For relatively small errors, only the parallel component of the complex measurement-error term will have a large influence on the amplitude. For this reason, the measurement variance for the normalized amplitude is doubled when inflating the variance (equation 3*a* [Disp-formula fd3]; Murshudov *et al.*, 1997 ▸), because only half of the complex variance is in this parallel direction. On the other hand, although the perpendicular component has a small influence on the amplitude, it consistently leads to an increase. Thus, the perpendicular component of the complex error increases the expected amplitude in the probability distribution, even though a random measurement error should not change the expected value of the measurement. Note that when σ_A_ is zero, (3*a*) reduces to a Wilson distribution for which the expected value of *E*_O_^2^ is 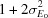. Consequently, the inflated-variance Rice function breaks the link between the downweighting by σ_A_ of the calculated normalized structure factor and the variance that is required to reinstate the total scattering. It is not obvious what function of the scalar error in amplitude should be used to inflate the variance to strike a balance between the competing problems of inflating to account for measurement error and deflating to reduce the errors thereby introduced into normalization. It is possible that some form of renormalization might improve the quality of this approximation, although we have not implemented this. Note that for centric reflections the distribution is one-dimensional, so the variance factor is 1 (3*b* [Disp-formula fd3]), but there is still a problem with data normalization.

### 1.2. MLI function   

**Figure 1:**
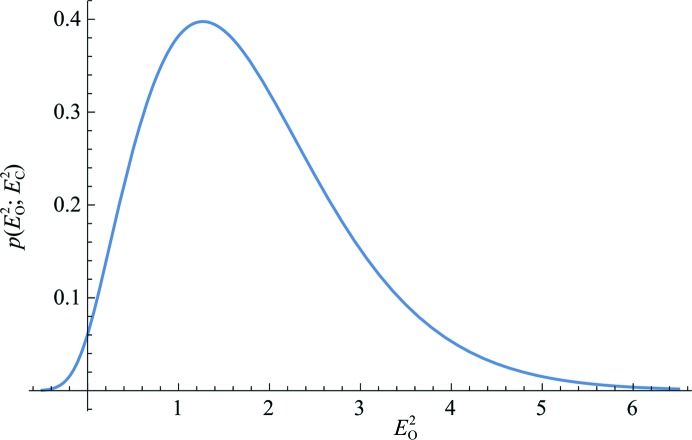
An example of the exact intensity likelihood function for the acentric case (6*b*), with *E*_C_ = 1.5, σ_A_ = 0.8 and 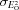 = 0.2. All figures were prepared using the program *Mathematica* (Wolfram Research, 2015 ▸).

Formulating likelihood functions in terms of intensities avoids a number of the problems described above. A change of variables provides the probability of the true normalized intensity given a model (equations 5*a* [Disp-formula fd5] and 5*b* [Disp-formula fd5] for the acentric and centric cases, respectively):
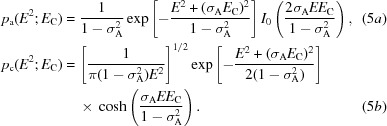



The MLI function is based on the simple statistical model that the observed intensity arises from the addition of a Gaussian measurement error to the true intensity. The effects of model and measurement errors can thus be combined by performing the convolution of the Rice function (expressed in terms of intensities; equation 5) and a Gaussian intensity-measurement error (6*a* [Disp-formula fd6]), yielding (6*b* [Disp-formula fd6]) and (6*c* [Disp-formula fd6]) for the acentric and centric cases, respectively:
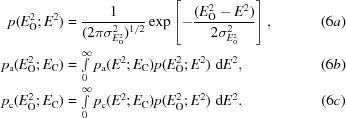



Fig. 1 ▸ shows, for the acentric case, the form of this probability distribution, which intrinsically allows the possibility of negative net intensities.

**Figure 2:**
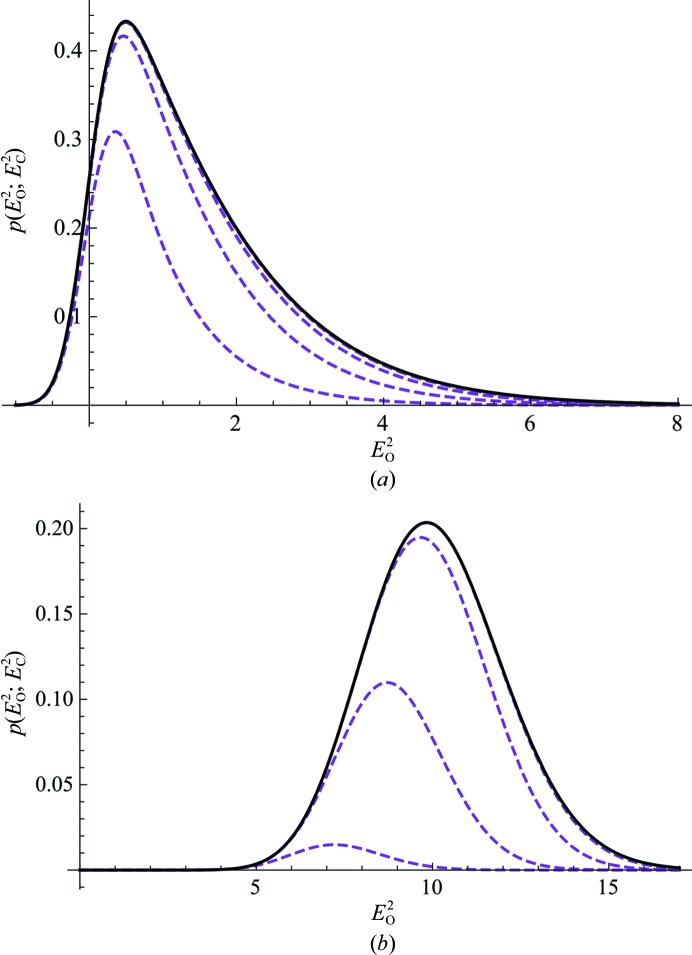
Illustration of the convergence of the MLI approximation (Pannu & Read, 1996 ▸) to the exact intensity likelihood function for the acentric case (6*b*). Approximations with increasing numbers of terms are shown as dashed magenta lines, while the exact function is shown in black. In both examples, the approximation with the largest number of terms is almost indistinguishable from the exact function. (*a*) Example with *E*_C_ = 2.0, σ_A_ = 0.4 and 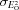 = 0.3. The dashed magenta curves show approximations with terms to order 0, 1, 2 and 3. (*b*) Example with *E*_C_ = 3.5, σ_A_ = 0.9 and 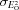 = 0.3. The dashed magenta curves show approximations with terms to order 40, 50, 60 and 70.

Unfortunately, there appears to be no analytical solution to the convolution integral for either the centric or acentric case. Calculation *via* numerical integration is prohibitively expensive for practical implementations, with many such integral evaluations for the likelihood function and its derivatives needed per reflection in the course of normal phasing. To circumvent this problem, the integrand can be rendered as a series approximation where the terms in the series can be integrated analytically. This numerical technique was used to develop the MLI target (also called MLF2 in Pannu & Read, 1996 ▸) for use in structure refinement in *CNS* (Brünger *et al.*, 1998 ▸). It is a viable approach when the series converges rapidly; however, as shown in Fig. 2 ▸, the MLI target has the serious disadvantage that it can be necessary to compute tens of terms in the series for convergence.

A more fundamental problem with the MLI target is that it does not lend itself to generalization to higher dimensions, which would be needed to develop likelihood targets for experimental phasing, since each correlation between structure factors included in the analysis requires another integration over the unknown phases and, in principle, over the measurement-error distribution. Only one of the phase integrals can be solved analytically so, in the acentric case, the Bessel-function term used in the series approximation is only present for one observation. Performing multi-dimensional numerical integration to deal with the other observations would lead to severe numerical instabilities and intractable computing requirements.

## 2. Intensity-based LLG function   

As described above, the deficiencies in the current treatments of experimental errors are numerous and varied. However, it is clear that working directly with intensities avoids the problems associated with conversion to amplitudes and has the advantage of keeping the target function closer to the actual observations. This is the strength of the MLI target. On the other hand, given the utility of the multivariate complex normal distribution (relating phased structure factors) in deriving crystallographic likelihood targets (Read, 2001 ▸, 2003 ▸; McCoy *et al.*, 2004 ▸), there are significant advantages in an approach that approximates intensity errors in some way as complex structure-factor errors, thus yielding targets based on Rice functions. Combining the strengths of the MLI target with the strengths of a target based on the Rice function would be ideal.

The inflated-variance Rice-function approximation was derived by starting from a Rice function for the probability of the true amplitude given the model and then adding the uncertainty arising from measurement error by increasing the size of the variance term in the Rice function. It is useful to consider a different approach in which the measurement error and the model error are treated as independent complex deviations from the true structure factor (treated as a dummy variable that connects the calculated and observed structure factors), instead of being added up in turn. In this approach, we deal separately with the model error and the measurement error and then combine their effects through their common relationship with the true structure factor.

The effects of model error are already well understood (equations 1 and 5), which leaves only the measurement error to be studied separately.

### 2.1. Log-likelihood-gain target function   

The mean-value theorem for integrals can be used to gain some insight into the properties of a Rice-function approximation for the effect of intensity-measurement error. The exact likelihood target is defined as the convolution integral of (6), which integrates over all possible values of the true (possibly normalized) structure-factor amplitude. The mean-value theorem tells us that there will be some value for *E* (or for *E* ^2^) in its range of integration (*i.e.* non-negative) that will allow a Rice function to be factored out of the integral, leaving the value of the integral unchanged. We will refer to this value of *E* as 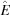, as shown in (7),
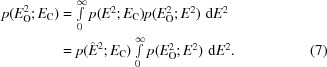



What we learn from (7) is that if the intensity-based likelihood is approximated by a Rice-function likelihood with some amplitude (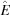) standing in for the observation, the Rice function itself will be at best proportional to the true intensity-based likelihood.

The proportionality constant given by the integral depends only on the observed intensity, not the calculated structure factor, so it will cancel out in either a likelihood ratio or a log-likelihood-gain (LLG) value. So what we might be able to approximate successfully using a Rice-function formula is the LLG and not the likelihood itself. The mean-value theorem, as expressed in (7), would provide a value for 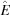 that corresponds to an exact solution in a particular circumstance, *i.e.* for particular values of *E*_C_ and σ_A_. For a practical treatment, we need an approximation that is good for a variety of *E*_C_ and σ_A_ values encountered throughout model optimization, but the goal should be an approximation for the LLG. An additional advantage of the LLG is that it is invariant to any transformation of the observations, as the Jacobian terms of such a transformation will cancel out in a likelihood ratio, so LLG scores for intensities and amplitudes are equivalent. The LLG also avoids the problem of dealing with reflections with an amplitude estimated as zero; the amplitudes in equations related to (1*a*) cancel out, so that the logarithm of zero does not appear in the calculations.

### 2.2. Modelling measurement error   

To develop a new approach to modelling the effect of measurement error as a complex error in the true structure factor, we start with the probability of the true normalized intensity given the observed normalized intensity: this is the French & Wilson (1978 ▸) posterior distribution for intensities and is obtained by using Bayes’ theorem (8) to manipulate distributions that we have determined.
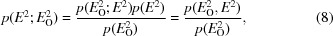



The probability of the observed normalized intensity in the denominator, which depends on the size of the experimental errors, is obtained by integrating the numerator over all possible values of the true normalized intensity, yielding (9*a*) and (9*b*) for the acentric and centric cases, respectively, 
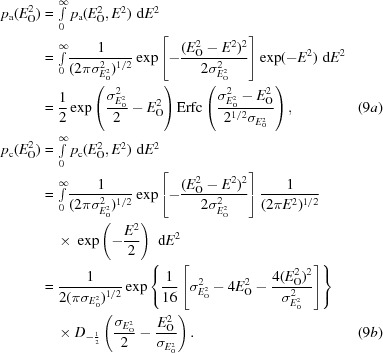



In these equations, Erfc is the complement of the error function and *D* is a parabolic cylinder function (Whittaker & Watson, 1990 ▸). These integrals, and most other new integrals in this work, were evaluated using the program *Mathematica* (Wolfram Research, 2015 ▸). The posterior probability for the true *E* value is obtained by a change of variable, giving (10*a*). In the Rice function defined in terms of normalized amplitudes, the conditional probability of one *E* value (the true *E* value in equation 1) given another *E* value (the calculated *E* value in equation 1) depends on the parameter σ_A_, which is the complex correlation between the two *E* values. To obtain Rice-function approximations to the probability of the true *E* value in (10*a*), we have to find values for two parameters that play roles analogous to *E*_C_ and σ_A_ in (1*a*) and (1*b*), which we will refer to as the effective *E* value (*E*_e_), representing information derived from the observed normalized intensity, and *D*_obs_, representing the reduction in correlation between observation and truth arising from experimental error. The form of these approximations is shown in (10*b*) for the acentric case and in (10*c*) for the centric case.
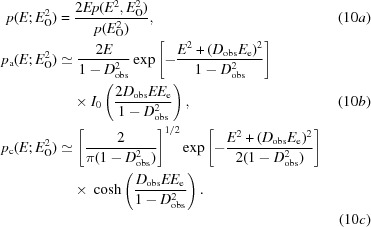



One could imagine many ways to find *E*_e_ and *D*_obs_ in (10*b*) and (10*c*) so that they approximate the function in (10*a*); for example, least-squares fitting. Ideally, the method should be analytical so that it is fast. Our approach is to match two moments of the distributions given by (10*a*) and by (10*b*) and (10*c*) to obtain values for these two variables by solving two simultaneous equations. Either the first and second moments can be matched or the second and fourth moments (which are the first and second moments of the normalized intensity).

The first two moments of (10*a*) are simply the posterior expected (normalized) amplitude and intensity defined by French & Wilson (1978 ▸). These expected values are obtained by integrating the product of the amplitude (or intensity) and its probability over all possible values from zero to infinity. Although French and Wilson proposed to determine these quantities by numerical integration, there are in fact analytical solutions to the expected value integrals, as there are for all of the other moments needed for this approach. For the acentric case, the first, second and fourth moments of the distribution in (10*a*) are given in equations (11*a*), (11*b*) and (11*c*), 
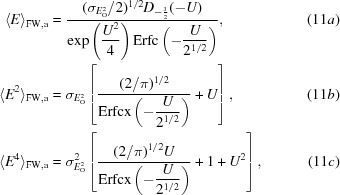
where
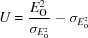
and
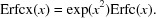



For the centric case, the first, second and fourth moments of the distribution in (10*a*) are given in (12*a*), (12*b*) and (12*c*), the integrals for which were evaluated based on equation #3.462.1 of Gradshteyn & Ryzhik (1980 ▸), 
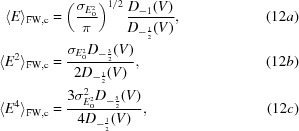
where
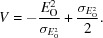



The first, second and fourth moments of the acentric Rice distribution (10*b*) are given in (13*a*), (13*b*) and (13*c*),
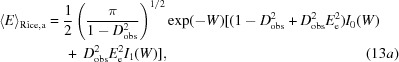
where
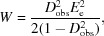


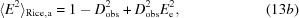


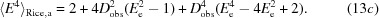



The first, second and fourth moments of the centric ‘Rice’ (Woolfson) distribution (10*c*) are given in (14*a*), (14*b*) and (14*c*),
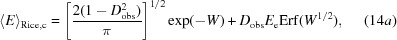
where
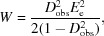


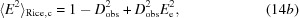


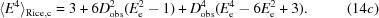



Appendix *A* [App appa] describes the algorithms used to determine the values of *E*_e_ and *D*_obs_ that match two pairs of moments. The results are very similar, whether the first and second or the second and fourth moments are matched, but the simplicity of the second-moment and fourth-moment equations for Rice distributions makes it easier to match these pairs of moments, with the additional advantage that there are analytical solutions. Appendix *B* [App appb] discusses solutions to numerical issues that arise in evaluating the parabolic cylinder functions required for these calculations.

## 3. Combining measurement and model errors   

To obtain a Rice-function-based LLG target that uses *E*_e_ and *D*_obs_ to represent the intensity measurement and its experimental error, what is needed is the probability of *E*_e_ given the calculated structure factor *E*_C_. We can obtain this by first constructing a joint probability distribution, in the form of a multivariate complex normal distribution, involving the phased structure factors **E**_e_ and **E**_C_, as well as the unknown true structure factor **E** as a dummy variable. For normalized structure factors, the covariance matrix is a correlation matrix with ones along the diagonal. The off-diagonal elements involving the true **E** are σ_A_ (for **E**_C_) and *D*_obs_ (for **E**_e_). For two random variables that differ in independent ways from a common variable, the correlation term is the product of their individual correlations to the common variable. This can be seen in the correlation matrix presented in (15), in which a superscript asterisk indicates the complex conjugate, 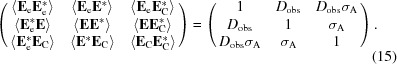



To obtain a correlation matrix describing the relationship between **E**_e_ and **E**_C_, the terms involving the dummy true **E** can simply be omitted to give (16),
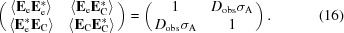



A probability distribution conditional on **E**_C_ can be defined based on the correlation matrix in (16); then, after a change of variables from complex **E**_e_ to amplitude and phase followed by integration over the unknown phase, likelihood functions can be defined in terms of *E*_e_ and *D*_obs_. These are shown for the acentric and centric cases in (17*a*) and (17*b*), analogous to (1*a*) and (1*b*),
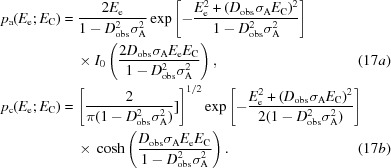



Taking account of the argument based on the mean-value theorem in (7), these equations are not expected to provide good approximations for the variation with *E*_C_ of the likelihood functions in (6*b*) and (6*c*). However, the corresponding LLG functions of (2) should provide much better approximations. The exact LLGs are obtained by dividing (6*b*) and (6*c*) by the likelihood for a null hypothesis (random-atom or uninformative model), given in (9*a*) and (9*b*), and then taking the logarithms of the ratios (or equivalently taking the differences of the logarithms), as shown in (18*a*) and (18*b*). 
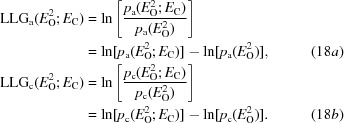



The LLGs for the Rice-function approximations, termed LLGI, are obtained by similar manipulations, with the results given in (19*a*) and (19*b*). Note that the likelihood for the null hypothesis is the Wilson distribution for *E*_e_, which can be obtained by setting σ_A_ to zero in the conditional distributions in (5). 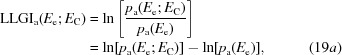
where
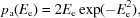


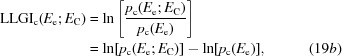
where
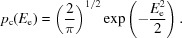



## 4. Implementation of log-likelihood-gain intensity targets   

Starting from observed diffraction data, there are a number of steps that must be carried out to use the new log-likelihood-gain intensity targets. When adapting programs that already use Rice-function likelihood targets, much of the underlying machinery can be preserved. The following discusses the changes that have been introduced in *Phaser* (McCoy *et al.*, 2007 ▸) to use intensity data for molecular-replacement calculations.

### 4.1. Normalization   

Even for data from crystals that diffract isotropically and do not possess translational NCS (tNCS), the uncertainty in the mean intensity introduced by measurement error can become significant at the resolution limit, which leads to imprecision in data normalization and in the application of the LLGI to measurement error.

In *Phaser*, the characterization of both anisotropy (McCoy *et al.*, 2007 ▸) and tNCS (Read *et al.*, 2013 ▸) has used likelihood functions based on the Wilson distribution, in which adjustable parameters describe the modulation of the expected intensity or Wilson variance, Σ*_N_*. However, the Wilson distribution does not account for the effect of intensity-measurement errors, which will broaden the distribution of observed intensities. It is therefore better to characterize anisotropy and tNCS with a likelihood target based on the probability distribution of observed intensities, including the effect of measurement errors. Such a likelihood target is derived from (9*a*) and (9*b*) by a change of variables based on *E*_O_^2^ = *I*_obs_/(∊Σ_*N*_), yielding (20*a*) and (20*b*),
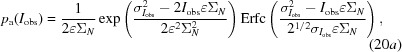


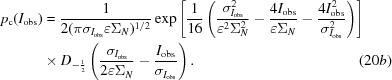



In the presence of extremely large measurement errors, the Wilson variance Σ*_N_* and the parameters describing its modulation can be poorly determined. The refinement of these parameters can be stabilized by adding prior information in the form of restraints to the *BEST* curve (Popov & Bourenkov, 2003 ▸). For instance, a data set prepared from diffraction patterns simulated by *MLFSOM* (James Holton, personal communication), which was used in testing new methods for SAD substructure determination (Bunkóczi *et al.*, 2015 ▸), was integrated to such a high resolution that the average intensity in some of the resolution shells is negative (although not significantly negative compared with the estimated errors). By using *BEST* curve restraints, even these data can be accommodated, although they contribute only minimally to likelihood targets.

### 4.2. Outliers   

Likelihood targets, including those used to characterize the intensity distribution, are very sensitive to the presence of outliers. An outlier test, similar in concept to one that ignores measurement errors (Read, 1999 ▸), can be based on a cumulative distribution function, defined generally in (21*a*). The cumulative distribution function for the acentric case, derived using (9*a*), is given in (21*b*). 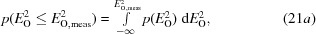


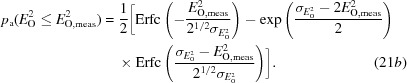



For the centric case, the cumulative distribution function is determined by numerical integration using the probability distribution defined in (9*b*). If the cumulative distribution function is less than some outlier probability threshold (such as 10^−6^), this implies that the observed net intensity is too negative to be consistent with the estimated measurement errors. On the other hand, if one minus the cumulative distribution function is less than the outlier probability threshold, this implies that the observed intensity is too large.

### 4.3. Dealing with data provided as amplitudes   

The methods described here will work most reliably with data provided as intensities. When data are provided in the form of amplitudes it is not clear how the intensities have been transformed to obtain them, so assumptions must then be made about the form of the transformation. Firstly, there is the question of whether the amplitudes have been processed using the French and Wilson algorithm. Such data can be detected by the fact that the prior Wilson distribution of intensities imposes an upper bound on the posterior standard deviations. In the limit of infinite intensity-measurement error, both the posterior amplitude and its standard deviation come from the Wilson distribution for amplitudes. We would need to know the variance for the Wilson distribution used in the prior for the French and Wilson algorithm to determine their values separately, but not their ratio. The minimum ratios of the French and Wilson posterior amplitude and standard deviation for acentric and centric data are given by (22*a*) and (22*b*).
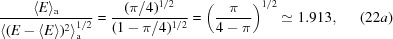


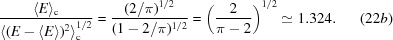



If there are no reflections with ratios significantly below these values (allowing for some rounding error in the storage of the data), then it is reasonable to assume that the data have been processed with the French and Wilson algorithm. For example, for PDB entry 3wrh (from the random sample of 100 entries discussed below) the minimum ratio is 1.35 for centric reflections and 1.97 for acentric reflections. Once the data have been recognized as such, the first two moments of the French and Wilson posterior distribution can be calculated from the posterior amplitude and its standard deviation, and these can then be used to compute *E*_e_ and *D*_obs_. However, it should be noted that this will only yield the same values that would have been obtained from the intensity data if the original French and Wilson treatment used the same Wilson variance (expected intensity) values. This is particularly un­likely to be true in the presence of translational noncrystallographic symmetry.

If lower values are found for the ratios of the amplitudes and their standard deviations, then some transformation other than the French and Wilson treatment must have been used. A number of ways to transform intensities to amplitudes have been proposed, and it is difficult to tell which has actually been used. We assume that the transformation given in (4) has been used and apply the inverse of this transformation to regenerate the intensities and their standard deviations. Note that any negative net intensities will either have been discarded or set to amplitudes of zero, so the information from these will have been lost or degraded. If a transformation other than (4) was used, then using the wrong inverse transformation will also degrade the quality of the intensity standard deviation.

There is an additional complication when dealing with data processed with the French and Wilson algorithm. An unbiased estimate of the original intensities, as required for normalization, cannot be obtained by squaring the amplitudes but rather by recovering the posterior expected intensity value by summing the squares of *F*_obs_ and 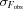.

### 4.4. Accounting for the effects of measurement errors in likelihood targets   

Once the data have been transformed to obtain *E*_e_ and *D*_obs_ values, Rice likelihood functions such as those given in (3) must be replaced by the LLGI target in (19*a*) and (19*b*). Similarly, derivatives with respect to any refineable parameters must also be revised. Given the close relationship between equations (3) and (19) [Disp-formula fd24], this part of the implementation should be relatively straightforward.

## 5. Results   

Separate tests have been carried out to determine how well the Rice-function approximations for measurement error alone represent the exact probability distributions, and how well the LLGI target approximates the exact LLG.

**Figure 3:**
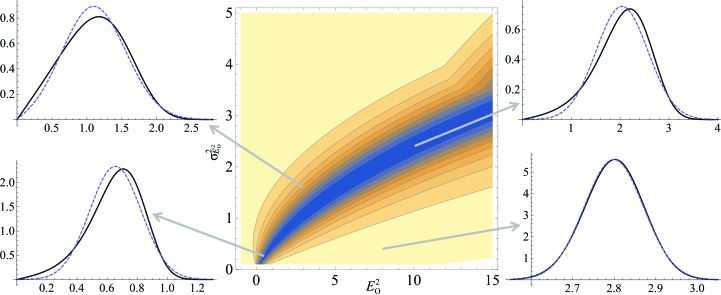
The central contour plot shows the correlation coefficient between the exact probability distribution for the true amplitude (10) with the effective *E* Rice-function approximation for the acentric case as a function of the observed normalized intensity and its estimated standard deviation. The darkest blue shading indicates regions where the correlation coefficient is greater than 0.990, and the contour lines are spaced by increments of 0.001, with the yellow shaded region indicating correlation coefficients greater than 0.999. The four line plots show comparisons between the exact probability distribution (black line) and the Rice-function approximation (dashed blue line) in four different regions of the space indicated by the tails of the arrows.

To test the quality of the Rice-function approximation for measurement error, a range of values for *E*_O_^2^ and 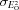 were explored and the exact probability distribution from (10) was compared with the Rice-function approximations computed by matching the second and fourth moments in (11) through (14) by computing the correlation between the two distributions. Fig. 3 ▸ presents a contour plot of the correlation values for the acentric case, along with comparisons of the exact and approximate distributions for points from regions with the highest and lowest correlations. The quality of the approximation for the centric case (not shown) is slightly lower overall, but is still acceptable.

In evaluating the quality of the LLGI, we wished to compare it not only with the exact LLG but also with the LLGs that would be obtained with the inflated-variance Rice-function approximations in current crystallographic programs, using different estimates for the observed amplitude and its standard deviation. The LLGs for the inflated-variance Rice-function approximations can be obtained as the log of the ratio between the likelihood calculated with (3) and the null hypothesis likelihood, obtained by setting σ_A_ to zero in (3).

**Figure 4:**
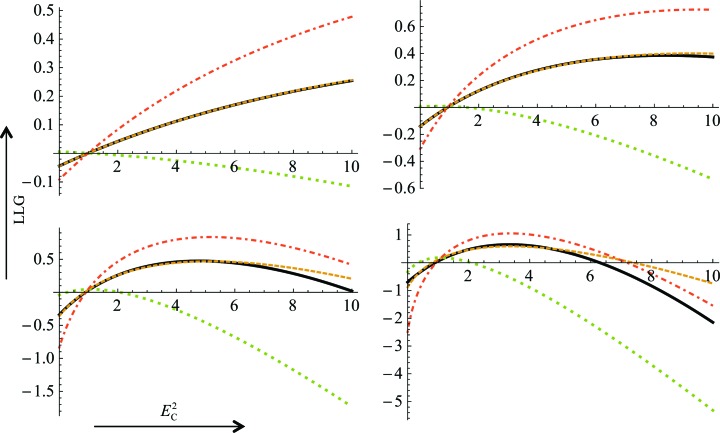
Comparisons of different approximations to the exact LLG for the acentric case as the quality of the model is varied. All four plots represent the case shown in the upper left line plot of Fig. 3 ▸, in which *E*_O_^2^ = 3.0 and 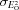 = 1.6. The exact probability distribution as a function of *E*_C_^2^ is shown as a black line, the LLGI approximation as a dashed orange line, the inflated-variance Rice-function approximation with French and Wilson estimates of the amplitude and its standard deviation as a dotted green line and the inflated-variance Rice-function approximation with estimates by simple variable transformation of the amplitude and its standard deviation as a dashed–dotted red line. The values of σ_A_ across the plots are 0.3 on the upper left, 0.5 on the upper right, 0.7 on the lower left and 0.9 on the lower right.

When the measurement error is relatively small, all of the approximations to the exact LLG are reasonably accurate (not shown). Fig. 4 ▸ provides an example showing that when the measurement error is relatively large, the LLG computed with LLGI provides much better results than the other approximations, particularly over the range of calculated structure factors that will be encountered most frequently during structure determination. Note that the French and Wilson estimates of the amplitude and its standard deviation actually give the worst results in the context of the inflated-variance Rice-function approximation because the posterior standard deviation is not an experimental error. As the experimental errors increase in size, the exact LLG and LLGI curves become very flat (because there is progressively less information in the data), but the inflated-variance Rice-function LLG continues to have a clear maximum, because the posterior standard deviation is bounded by the standard deviation of the Wilson distribution. This could provide an explanation for reports that maximum-likelihood refinement gives better results on pruned data, even applying ellipsoidal truncation in the case of severe anisotropy (Strong *et al.*, 2006 ▸).

## 6. Discussion   

In essence, the LLGI function for accounting for experimental errors in log-likelihood-gain target functions starts by finding values for two parameters, the effective *E* value (*E*_e_) and *D*_obs_, which can stay constant throughout a phasing or refinement calculation. *E*_e_ serves the role of the observed normalized amplitude and, when the σ_A_ values characterizing the effects of model error are multiplied by *D*_obs_, the resulting Rice LLGI function provides an excellent approximation to a true LLG that could only be evaluated by numerical integration. Even though LLGI is cast in terms of a function that (for the acentric case) implies complex errors, it is developed as an approximation to a log-likelihood gain based on the MLI target. As a result, the underlying statistical model is shared with the MLI target.

Note that if the observed intensity data are drawn from a Wilson distribution, it would be possible to refine some model of the observation errors (for example, a scale factor or a linear transformation) to give better agreement with likelihood targets based on (20), using the fact that there is an analytical relationship between the intensity errors and the *E*_e_ and *D*_obs_ parameters. This could supplement existing methods to adjust error models based on agreement among replicate measurements (Evans & Murshudov, 2013 ▸); in principle, better error models could be obtained as other information improves, such as from an atomic model. However, such an approach would have to be used with caution, as data from crystals with pathologies such as twinning would not obey the assumed Wilson distribution.

The LLGI function can be used to account for the effect of measurement errors in any applications that use Rice likelihood functions by first analysing the intensity data to produce *E*_e_ and *D*_obs_ values and then replacing the likelihood targets based on (1) with the modified equations (17). Applications include σ_A_ estimation (Read, 1986 ▸), which is used to estimate phase probabilities, likelihood-based molecular replacement (McCoy *et al.*, 2007 ▸) and structure refinement (Pannu & Read, 1996 ▸; Murshudov *et al.*, 1997 ▸; Bricogne & Irwin, 1996 ▸; Afonine *et al.*, 2012 ▸). This approach can also be generalized to the collections of structure factors required for experimental phasing, and preliminary work has been carried out on applying it to single-wavelength anomalous diffraction (SAD) phasing.

In macromolecular crystallography it has become standard practice to apply the French and Wilson algorithm to the merged intensities and to use these amplitudes and standard deviations in all downstream crystallographic calculations. For example, in the *CCP*4 suite (Winn *et al.*, 2011 ▸) this calculation is performed by *CTRUNCATE* as a default procedure after data scaling with *AIMLESS* (Evans & Murshudov, 2013 ▸) through the *CCP*4*i* interface. The standard deviation obtained by this approach is thus used as an estimate of the experimental error throughout likelihood-based phasing and refinement, a purpose for which it was not intended.

Unfortunately, the original intensity information is lost more often than not on deposition in the worldwide Protein Data Bank (Berman *et al.*, 2003 ▸). A snapshot of current practice by depositors was obtained by randomly selecting 100 of the 2769 X-ray structures released by the wwPDB in the first four months of 2015. Of these, 39 contained intensity data but 61 contained only amplitudes with no intensity data. Of these 61, 54 contained amplitudes that had apparently been produced by the French and Wilson algorithm, as detected by the test described in §[Sec sec4.3]4.3. The remaining seven contained amplitudes that had been produced by some other transformation. Given the inevitable loss of information by the transformation to amplitudes, we recommend that all crystallographers should include the original intensity data in future depositions in the wwPDB, possibly in addition to amplitudes if these were used for refinement.

The use of the French and Wilson algorithm depends on the expected intensities, which can be estimated more precisely when the anisotropy has been modelled and/or the expected intensity factors from tNCS have been determined. Without correction for non-isotropic systematic variations in intensity, the posterior amplitudes and intensities that emerge from the French and Wilson treatment are systematically overestimated for the systematically weak data, because the prior expectation is for an intensity that is too large. As a result, measures of anisotropy and tNCS tend to be damped when data processed with the French and Wilson algorithm are analysed. The French and Wilson estimates of *F* and σ_*F*_ should be updated as knowledge of the anisotropy and tNCS improves in the process of structure solution. Any conversion to amplitudes using the French and Wilson algorithm should be carried out as required, and not kept invariant for the entire structure-solution process. In the same way, the calculation of *E*_e_ and *D*_obs_ should be carried out when required and the results should not be stored.

The LLGI function is the template for likelihood targets working with intensities and their errors throughout the structure-solution process. However, work needs to be performed to investigate how one should account for measurement errors in other methods based on structure-factor probabilities. Although the *E*_e_ and *D*_obs_ parameters provide an excellent approximation to likelihood targets, the use of *D*_obs_ in phase probability equations would ascribe a role to the perpendicular component of the assumed complex measurement error, leading to a pessimistic view of phase errors. In the limit of infinite measurement error, *D*_obs_ will be zero, leading to a figure of merit of zero, even though the accuracy of the calculated phase depends on the overall accuracy of the model, not of a particular measurement. The downweighting of structure factors for uninformative measurements would fortuitously reduce potential model bias, but further work will be needed to determine the optimal procedures for map calculation.

In this work, we have assumed that the standard deviations of the observed intensities have been estimated accurately, but this is a difficult problem (Phil Evans, personal communication). By providing a method that will make good use of measurement-error estimates, we hope to have provided a further incentive to improve the accuracy of these estimates.

## 7. Availability   

LLGI has been implemented and tested in *Phaser*. Releases from v.2.5.7 will accept intensities in preference to amplitudes for molecular replacement, and a future version will accept intensities in preference to amplitudes for SAD phasing. Please refer to the documentation (http://www.phaser.cimr.cam.ac.uk) for details.

All of the code required to compute *E*_e_ and *D*_obs_ for both acentric and centric cases, given the observed intensity, its estimated standard deviation and an estimate of the Wilson variance for the scattering power, has been contributed to the *cctbx* library (Grosse-Kunstleve *et al.*, 2002 ▸), where it is available in open-source form for use in other programs. The ancillary code needed to compute the parabolic cylinder functions was adapted, with permission, to C++ from the Fortran program *mpbdv.for* (Zhang & Jin, 1996 ▸). This has been contributed to the *scitbx* library distributed with *cctbx*.

## Figures and Tables

**Table 1:** Terms used in this paper

*E*	True normalized structure-factor amplitude
*E*_O_	Observed normalized structure-factor amplitude
*E*_C_	Calculated normalized structure-factor amplitude
*E*_e_	‘Effective’ *E*, used in the Rice-function approximation to the intensity probability distribution
*D*_obs_	Luzzati-style *D* factor, encoding correlation between true and ‘effective’ *E* values in the Rice-function approximation to the intensity probability distribution
*σ*_A_	Parameter describing the correlation between the true and calculated *E* values
*I*_obs_	Observed intensity
〈*E^n^*〉_FW,a_	Expected value of *E^n^* in the French and Wilson distribution, acentric case
〈*E^n^*〉_FW,c_	Expected value of *E^n^* in the French and Wilson distribution, centric case
〈*E^n^*〉_Rice,a_	Expected value of *E^n^* in the Rice distribution, acentric case
〈*E^n^*〉_Rice,c_	Expected value of *E^n^* in the Rice distribution, centric case
*D*_ν_(*x*)	Parabolic cylinder function of order ν
Erf(*x*)	Error function
Erfc(*x*)	Complement of the error function
*I*_*n*_(*x*)	Modified Bessel function of order *n*

## Appendix A Evaluation of *E*_e_ and *D*_obs_ by the method of moments

As discussed in §[Sec sec2.2]2.2, Rice-function approximations of the probability distribution for the true normalized structure factor given the observed intensity (10) can be obtained by finding values of the parameters *E*_e_ and *D*_obs_ that make either the first two moments or the second and fourth moments of the two distributions equal by solving two simultaneous equations. Note that the second and fourth moments are the first two moments of the distribution of the normalized intensity.

### A1. Matching the first two moments

The two simultaneous equations can be set up by taking the values from the first two moments for the exact distribution, in (11) for the acentric case or (12) in the centric case, and solving two simultaneous equations for the Rice-function moments in (13) [Disp-formula fd14] [Disp-formula fd15] for the acentric case or (14) [Disp-formula fd17] [Disp-formula fd18] for the centric case. We have not been able to find analytical solutions for the equations defined by the first two moments. However, note that the equation for the second moment (which is the same for the Rice functions for both the acentric and centric cases) can be solved for *E*_e_^2^,

This can be substituted into the equation for the first moment to obtain single equations in one unknown for the acentric case (24*a*) and the centric case (24*b*),

where

and

where

The exponential Bessel function (e*I_n_*) is used in (24*a*) to avoid overflow for large arguments. Solutions to (24*a*) or (24*b*) can be obtained by a line search. Because these equations are defined in terms of the squares of *E*_e_ and *D*_obs_, there are generally four solutions. To find only the solution corresponding to real values of both *E*_e_ and *D*_obs_, the line search can be constrained to positive values of *D* ^2^_obs_ above the value giving a minimum in either (24*a*) or (24*b*). The solution is well defined for physically reasonable values of the normalized intensity and its standard deviation, but it can be poorly defined for improbable values. In such cases, the best approximation of the Rice function will be similar to the Wilson distribution; a solution of good quality can be obtained simply by setting *D*_obs_ to 0.05 and determining *E*_e_ using (23). Under some circumstances this will yield a very large value of *E*_e_, in which case *E*_e_ can be set to some maximum, such as 10, and (23) can be solved for the corresponding *D*_obs_. This simple prescription has been validated over a very wide range of input values in a plot similar to Fig. 3 ▸ (omitting points that would be identified as clear outliers by equations 21) [Disp-formula fd28].

### A2. Matching the second and fourth moments

This approach is more straightforward because the simplicity of the fourth-moment equations compared with the first-moment equations allows analytical solutions of the pairs of equations. For both the acentric and centric cases, there are two solutions in terms of *E*_e_^2^ and *D* ^2^_obs_, one of which yields positive values for physically reasonable inputs. This solution provides (25*a*) and (25*b*) for the acentric case and (26*a*) and (26*b*) for the centric case.

There are combinations of normalized intensity and standard deviation that generate second and fourth moments for which these solutions yield negative values or excessively large effective intensities. All such combinations correspond to outliers or extremely large experimental errors. In such cases, good Rice-function approximations can nonetheless be obtained with moderate arguments by following the same prescription as described in §[Sec seca1] *A*1.

## Appendix B Computing parabolic cylinder functions for large arguments

The parabolic cylinder functions are well behaved for moderate arguments, but there are problems with underflow and overflow for the large positive and negative arguments that can be encountered with real data. In such cases, a ratio or product of functions factored out of the overall function can be well behaved. These ratios or products, in turn, can be evaluated accurately with asymptotic approximations. One example is the parabolic cylinder function of order minus one-half. When this is appropriately scaled (using a scaling function similar to those suggested by Gil *et al.*, 2006 ▸), an asymptotic approximation can be obtained for either large negative arguments (equation 27*a* [Disp-formula fd38], applicable to *x* < −16) or large positive arguments (equation 27*b*, applicable to *x* > 16),

One more example is the ratio of parabolic cylinder functions of order minus one and minus one-half. Asymptotic approximations can be obtained for large negative arguments (equation 28*a* [Disp-formula fd40], applicable for *x* < −17.5) or large positive arguments (equation 28*b* [Disp-formula fd41], applicable for *x* > 17.5),

Other approximations can be found in the source code in the *cctbx* library.
